# A comparison of short-term outcomes between robot-assisted percutaneous vertebroplasty and manual percutaneous vertebroplasty in the treatment of osteoporotic thoracolumbar vertebral compression fractures

**DOI:** 10.3389/fsurg.2025.1678914

**Published:** 2025-10-14

**Authors:** Hang Lin, Kun Li, Zhibin Zhang, Abuduwupuer Haibier, Tuerhongjiang Abudurexiti

**Affiliations:** 1 Spinal Surgery, Sixth Afliated Hospital of Xinjiang Medical University, Urumqi, Xinjiang Uygur, China; 2 Xinjiang Medical University, Urumqi, Xinjiang Uygur, China

**Keywords:** robot, robot-assisted navigation, percutaneous vertebroplasty, osteoporotic thoracolumbar vertebral compression fracture, cement leakage

## Abstract

**Objective:**

The study aims to evaluate the clinical efficacy of robot-assisted vs. manual percutaneous vertebroplasty in managing osteoporotic thoracolumbar vertebral compression fractures.

**Methods:**

Based on the inclusion criteria, 111 patients who received unilateral percutaneous vertebroplasty (PVP) surgery at the Sixth Affiliated Hospital of Xinjiang Medical University between September and December 2023 were retrospectively reviewed. These patients were categorized into two groups according to surgical technique: the robotic-assisted group (*n* = 43) and the manual group (*n* = 68). The study compared demographic and clinical parameters between the groups, including age, sex, Body Mass Index(BMI), medical history (hypertension, diabetes, respiratory diseases, endocrine disorders), affected spinal segments, Visual Analogue Scale(VAS) and Oswestry Disability Index (ODI) scores, vertebral height restoration, Cobb angle, operative duration, cement volume, cement leakage, intraoperative blood loss, postoperative hospital stay, and total hospitalization costs.

**Results:**

No statistically significant differences were observed between the robotic-assisted and manual groups regarding age, gender, BMI, affected vertebral segments, medical history, preoperative/postoperative VAS and ODI scores, vertebral height restoration, Cobb angle, cement volume, total hospitalization costs, blood loss, or postoperative hospital stay (*P* > 0.05). However, significant differences were found between the two groups in both operative time and cement leakage rates (*P* < 0.05).

**Conclusion:**

Manual percutaneous vertebroplasty demonstrates superior operative time compared to robot-assisted percutaneous vertebroplasty. However, the robotic approach offers the advantage of reduced cement leakage, which enhances procedural safety and decreases postoperative complications to some degree. Furthermore, as surgeons gain proficiency with the robotic system, operative time can be further reduced. This technology warrants continued refinement and represents a promising direction for future development.

## 1 Introduction

With the aging global population, osteoporotic vertebral compression fractures (OVCFs) are increasingly prevalent, accounting for over 8.9 million cases annually and emerging as a significant public health concern (1). This condition frequently affects elderly patients, often presenting with multiple fractures, and can be managed through either conservative or surgical approaches (2). Conservative management typically involves analgesics, bracing, and bed rest to enhance functional recovery and reduce the risk of future vertebral fractures. However, these methods have restricted effectiveness and may lead to adverse effects such as thrombosis and pulmonary infections. While generally safe, conservative treatment often fails to deliver optimal clinical results (3). In contrast, minimally invasive spinal surgery, particularly PVP, has gained widespread use for OVCF. PVP, commonly performed via a unilateral approach, stabilizes the vertebral body by injecting bone cement into the affected vertebrae. Polymethylmethacrylate (PMMA) remains the preferred cement due to its chemical stability, affordability, and strong biomechanical properties. Studies have demonstrated PVP's effectiveness in alleviating pain, preserving vertebral integrity, and preventing complications associated with prolonged immobility (4). Nevertheless, despite being a standard minimally invasive technique, PVP carries risks such as cement leakage, nerve or spinal cord damage, and adjacent vertebral fractures (2). Robot-assisted bone cement injection enhances puncture accuracy, improves cement distribution, lowers complication rates, and reduces leakage incidence. This study compares robot-assisted and manual PVP for OVCF, offering data-driven insights and clinical recommendations for selecting the optimal surgical approach.

## 2 Subjects and methods

### 2.1 Design

Retrospective comparative test.

### 2.2 Date and place

The study was carried out at the Spine Surgery Department of Xinjiang Medical University's Sixth Affiliated Hospital during a three-month period from September to December 2023.

### 2.3 Object

This investigation enrolled 111 consecutive cases (31 males, 80 females) diagnosed with osteoporotic thoracolumbar compression fractures at the Spine Surgery Department of Xinjiang Medical University's Sixth Affiliated Hospital from September to December 2023. All subjects underwent unilateral PVP, with vertebral fractures distributed across: T6 (2 cases), T7 (1 case), T8 (4 cases), T9 (4 cases), T10 (2 cases), T11 (7 cases), T12 (18 cases), L1 (29 cases), L2 (21 cases), L3 (14 cases), L4 (6 cases), and L5 (3 cases). The surgical interventions comprised 43 robot-assisted PVP procedures and 68 conventional manual PVP operations. Ethical approval for the study was granted by the Ethics Committee of the Sixth Affiliated Hospital of Xinjiang Medical University.

### 2.4 Inclusion criteria

(1) All sequential patients receiving first-time unilateral PVP procedures at the Spine Surgery Unit of Xinjiang Medical University's Sixth Affiliated Hospital during the September-December 2023 period; (2) Imaging-verified acute osteoporotic vertebral fractures (confirmed by x-ray, CT, and MRI examinations) supported by bone mineral density test results; (3) Involvement restricted to one vertebral body; (4) Complete medical records documenting at least six months of postoperative follow-up.

### 2.5 Exclusion criteria

(1) Preoperative manifestations of nerve root injury; (2) Comorbid severe neurological/psychiatric disorders or systemic illnesses impairing pain assessment compliance; (3) Pathological fractures secondary to neoplastic or infectious etiologies; (4) Prior spinal surgical intervention; (5) Multisegmental vertebral fractures.

### 2.6 Subgroup

Machine group: robot-assisted percutaneous vertebroplasty.

Manual group: manual percutaneous vertebroplasty.

### 2.7 General information

This retrospective study examined 111 consecutive unilateral PVP cases performed at Xinjiang Medical University's Sixth Affiliated Hospital from September to December 2023. Patients were categorized by surgical approach into two comparative groups: the Machine group (43 patients, average age 70.76 ± 10.12 years) and the manual group (68 patients, mean age 69.16 ± 8.47 years).

### 2.8 Equipment information

The information of the navigation and positioning equipment for spinal surgery is as follows (see Table 1).

**Table 1:** Robotics.

Device name	Spinal surgery navigation and positioning equipment
Production companies	Suzhou Casting Robotics Co.
Model	PSIS-A
product batch number	20230301-18
Registration certificate number	Su drug supervision equipment production licence 20200249

### 2.9 Surgical methods

#### 2.9.1 Preoperative preparation

All patients received thorough preoperative assessments before vertebroplasty, consisting of hepatic and renal function analyses, full blood workup, bone density evaluation, serum markers of bone metabolism, in addition to diagnostic imaging with MRI, CT scans, and radiographs.

#### 2.9.2 Surgical method of PVP

A single surgical team performed all procedures in both study groups. Patients were uniformly positioned prone under local anesthesia and received unilateral pedicle puncture. The vertebroplasty procedures utilized Shandong Guanlong Medical Supplies Co., Ltd. equipment along with Heraeus Medical GmbH bone cement. In the robotic-assisted cases, Suzhou Casting Robotics Co.'s spinal surgery navigation and positioning system was employed.

##### 2.9.2.1 Machine group

All procedures began with patients in the prone position, using C-arm fluoroscopy to locate the target vertebrae. After standard surgical preparation (disinfection and draping), robotic assistance facilitated accurate pedicle targeting. Post-robotic data processing, local anesthesia was induced at the puncture site with 2% lidocaine hydrochloride (Tianjin Jinyao Group Hubei Tianyao Pharmaceutical Co., Ltd.; 0.1 mg). Once anesthesia took effect, a 0.5 cm skin incision was created, allowing robotic-guided insertion of an expander through the pedicle into the anterior two-thirds of the vertebral body. Fluoroscopic verification ensured proper placement before controlled bone cement injection, with real-time monitoring of cement dispersion. Following cement hardening, the delivery needle was withdrawn, and a sterile dressing was applied. Aseptic protocols were rigorously observed, and intraoperative fluoroscopic records were systematically stored. Post-procedure, patients were transported to the ward on a flatbed.

##### 2.9.2.2 Manual group

The surgical protocol involved positioning all patients prone, with C-arm fluoroscopy used to locate target vertebrae. After standard sterile preparation (disinfection and draping), local anesthesia was achieved using 2% Lidocaine Hydrochloride Injection (Tianjin Jinyao Group Hubei Tianyao Pharmaceutical Co., Ltd.) at the planned puncture site. Surgeons then employed a unilateral transpedicular approach, carefully adjusting the bone cement puncture needle under continuous fluoroscopic guidance to achieve optimal positioning in the anterior third of the vertebral midline. Real-time fluoroscopy monitored the injection of polymethylmethacrylate bone cement (2.1–10.0 mL), with close attention to dispersion patterns. Post-polymerization, the delivery system was withdrawn and the entry point dressed sterilely, with strict maintenance of aseptic conditions. Standard documentation included intraoperative fluoroscopic images, and patients were moved to recovery via flatbed transport.

#### 2.9.3 Postoperative management

Postoperative care protocol after vertebroplasty involved: (1) 4–8 h of bed rest followed by ambulation with lumbar support bracing; (2) Standard osteoporosis management consisting of 12-month oral calcium carbonate D (600 mg/day) plus vitamin D supplementation; and (3) Intravenous zoledronic acid (Yangzijiang Pharmaceutical Group Sichuan Hairong Pharmaceutical Co., Ltd; 5 mg/100 mL; NMPA H20183098) administered on postoperative day 2. To mitigate potential bisphosphonate complications, patients received oral NSAIDs and intravenous 0.9% sodium chloride solution (Sichuan Kelun Pharmaceutical Co., Ltd; 500 mL; NMPA H51021158) starting 24–48 h before infusion. Surgical outcomes were evaluated through postoperative radiographic assessment.

### 2.10 Evaluating indicator

Patient age, gender, BMI, past history, fractured vertebrae, VAS score, ODI score, vertebral recovery height, Cobb's angle, operative time, cement dosage, cement leakage, haemorrhage, postoperative hospital stay, and total hospital costs.

**Figure 1:**
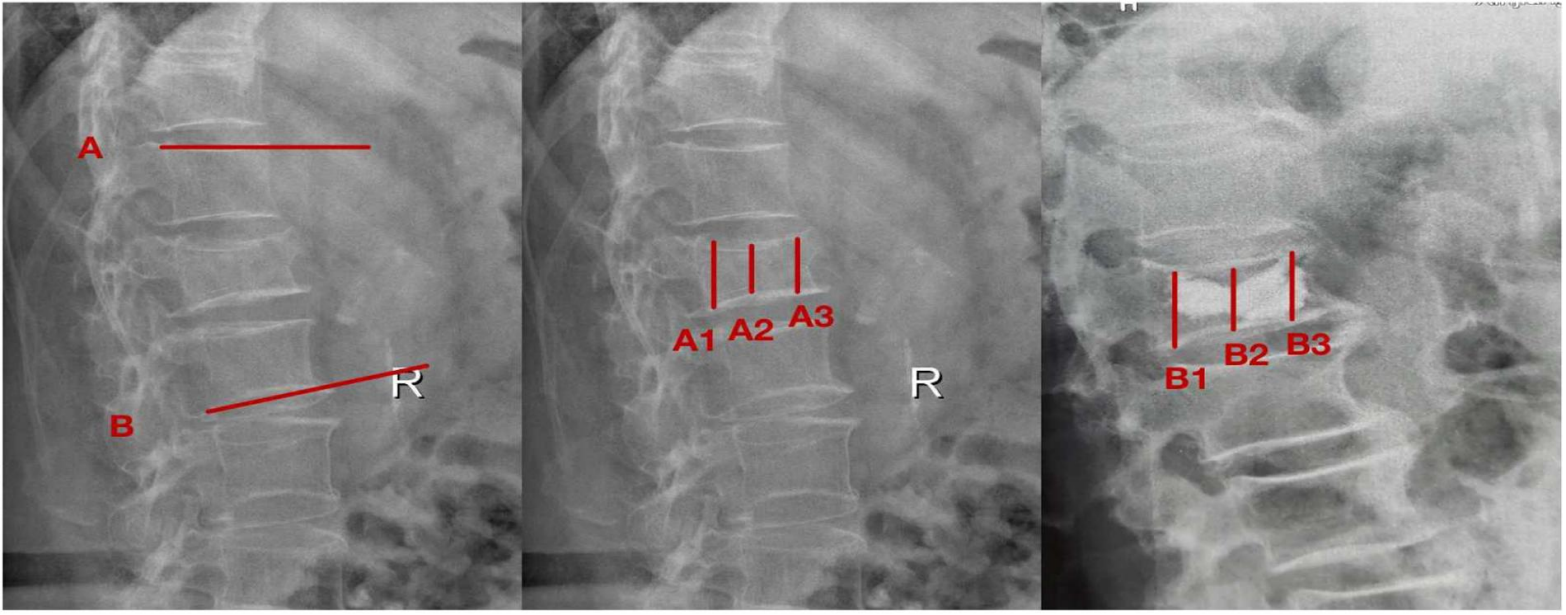
Measurement of imaging parametersafter vertebroplasty. The reference lines were defined as follows: Line A runs parallel to the superior endplate of the intact vertebra immediately cranial to the fracture, while Line B parallels the inferior endplate of the adjacent intact vertebra caudal to the fracture. Preoperative measurements included: A1 (posterior vertebral margin), A2 (fractured vertebral height), and A3 (anterior vertebral margin). Corresponding postoperative measurements comprised: B1 (posterior margin), B2 (restored vertebral height), and B3 (anterior margin).

1. The local kyphosis angle was calculated by measuring the angular intersection between two defined reference lines: Line A (drawn parallel to the superior endplate of the intact vertebra immediately cranial to the fracture) and Line B (positioned parallel to the inferior endplate of the adjacent intact vertebra caudal to the fracture), as illustrated in Figure 1.
2. Postoperative radiographic evaluation included vertebral height measurement via plain films, assessing three parameters: (1) projected original height (determined from the mean of neighboring intact vertebrae), (2) pre-intervention fracture height [A2, calculated as the average of anterior [A3] and posterior [A1] border measurements], and (3) post-procedural restored height [B2, derived from anterior [B3] and posterior [B1] margin averages]. Height restoration percentage was computed using the formula: [(B2-A2)/projected original height] × 100%, as depicted in Figure 1.

### 2.11 Statistical method

Statistical analysis was performed in SPSS version 26.0. The analysis included: (1) patient baseline characteristics (age, gender, BMI value); (2) clinical background and fracture characteristics (affected vertebral level); and (3) treatment outcomes (VAS score, ODI score, vertebral height restoration, and Cobb angle measurement). Similarly, surgical parameters were also evaluated, including operation duration, amount of bone cement injected, incidence of leakage, intraoperative blood loss, length of postoperative hospital stay, and overall treatment cost. Measurement data were assessed for normality using the Shapiro–Wilk test. Data conforming to a normal distribution are expressed as x ± s, while non-normally distributed data are described as M (Q1, Q3). For measurement data collected preoperatively and at 1 month, 3 months, 6 months postoperatively, and the final follow-up, repeated measures analysis of variance was used (applying the Greenhouse-Geisser correction when the sphericity test was not met). If the differences were statistically significant, pairwise comparisons between different time points were further conducted using the Bonferroni method. Comparisons of measurement data between preoperative and the final follow-up were performed using paired *t*-tests or the Wilcoxon signed-rank test. A two-sided test was used with a significance level of α = 0.05.

## 3 Results

### 3.1 Analysis of the number of participants

The study enrolled 111 patients undergoing unilateral PVP, stratified by surgical technique into two cohorts: 43 cases in the robot-assisted group and 68 in the manual group. All participants completed the study protocol without attrition, with complete data available for outcome analysis.

### 3.2 Test flow chart

**Figure 2:**
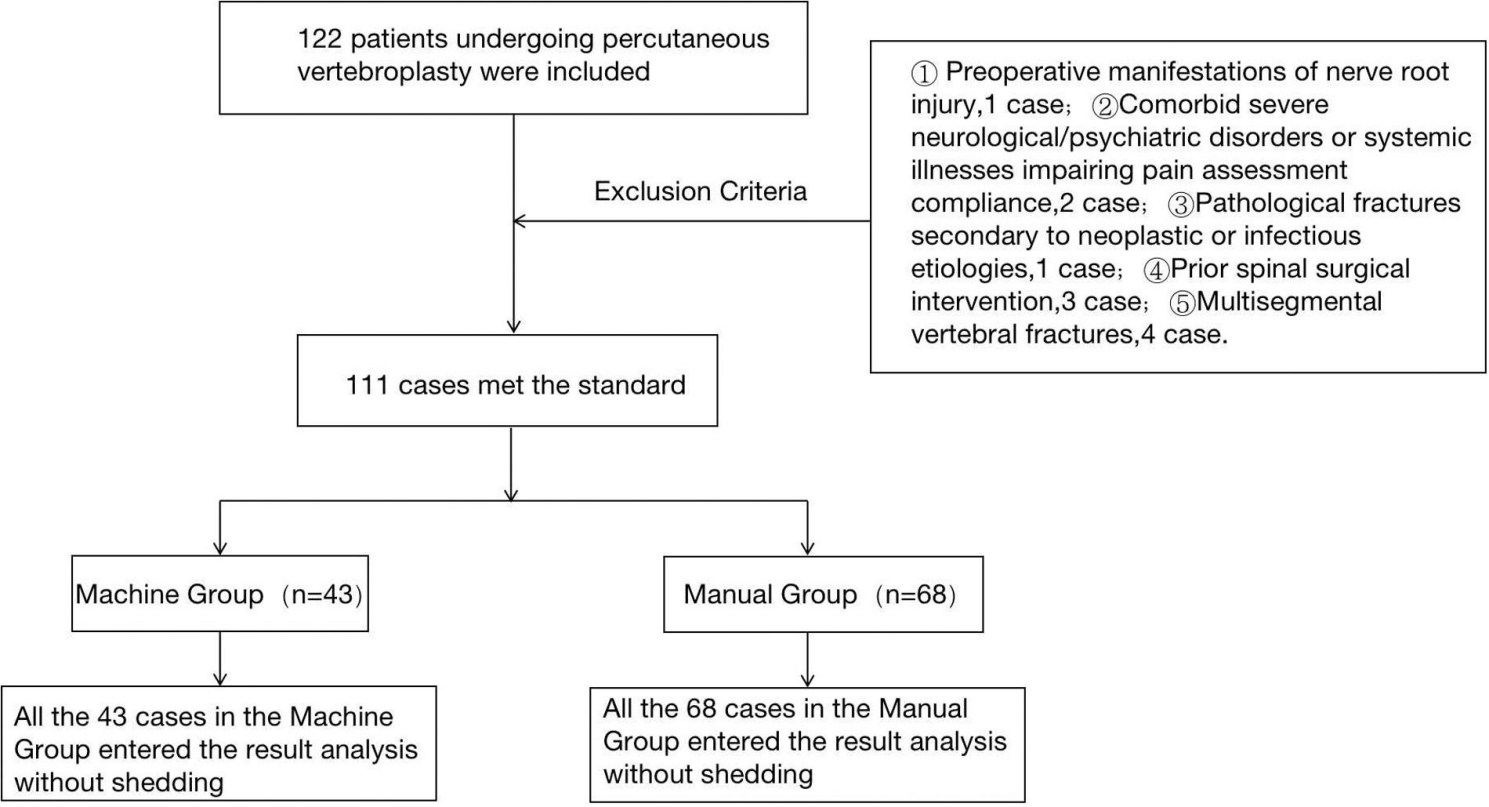
Flow chart of test grouping.

The flow chart of the two groups is shown in Figure 2.

#### 3.2.1 Preoperative general data of patients in the two groups

The robotic-assisted and manual PVP groups demonstrated comparable baseline characteristics, with no statistically significant differences in age, gender distribution, BMI, affected vertebral levels, medical history, preoperative VAS scores, or preoperative ODI scores (*P* > 0.05), see Table 2.

**Table 2:** Basic preoperative data of the two groups.

Items	Machine group (*n* = 43)	Manual group (*n* = 68)	*X^2^*/t	*p*
Age ($\bar{X}\pm S$)	70.76 ± 10.12	69.16 ± 8.47	3.074	0.389
Sex (*n*, male/female)	8/35	23/45	3.031	0.082
BMI ($\bar{X}\pm S$, kg/m^2^)	24.05 ± 4.00	24.53 ± 5.13	0.676	0.579
Fracture site (*n*/%)			3.758	0.053
C	10	28		
L	33	40		
Hypertension	18	35	0.975	0.323
Diabetes	17	39	3.346	0.067
Respiratory disease (*n*/%)	5	12	0.736	0.391
Endocrine system disease (*n*/%)	11	25	1.503	0.220
smoking (*n*/%)	5	14	1.491	0.222
drinking (*n*/%)	8	12	0.016	0.898
Preoperative VAS	6.79 ± 1.24	7.07 ± 1.38	0.718	0.267
Preoperative ODI	71.41 ± 3.94	69.61 ± 7.92	6.001	0.169

### 3.3 Comparison of postoperative VAS scores and ODI scores

No statistically significant differences were observed in postoperative low back pain VAS scores or ODI scores between the robotic-assisted and manual PVP groups (*P* > 0.05), see Table 3.

**Table 3:** Postoperative VAS and ODI scores in both groups.

Items	Follow-up time	Machine group (*n* = 43)	Manual group (*n* = 68)	t	*P*
Low back VAS score ($\bar{X}\pm S$, score)	1 month	3.86 ± 1.03^a^	3.60 ± 1.54^a^	0.965	0.338
	3 month	2.76 ± 1.32^a^	2.67 ± 1.27^a^	0.358	0.721
	6 month	1.41 ± 1.19^a^	1.69 ± 0.93^a^	−1.343	0.183
Low back ODI score ($\bar{X}\pm S$, %)	1 month	45.79 ± 6.98^a^	43.01 ± 9.27^a^	1.790	0.076
	3 month	28.60 ± 4.62^a^	27.41 ± 8.64^a^	0.832	0.407
	6 month	20.65 ± 2.32^a^	21.80 ± 6.47^a^	−1.126	0.263

Compared with preoperatively.

a *P* < 0.05: The continuous value was given as the mean and the standard deviation.

### 3.4 Comparison of vertebral recovery height and Cobb angle

Radiographic assessment revealed no statistically significant differences in vertebral height restoration or Cobb angle measurements between the robotic-assisted and manual PVP groups when comparing preoperative and postoperative day 1 imaging results (*P* > 0.05), see Table 4.

**Table 4:** Comparison of x-ray vertebral recovery height and Cobb angle before and 1 day in the two groups.

Items	Follow-up time	Machine group (*n* = 43)	Manual group (*n* = 68)	*t*	*P*
Vertebral height (mm)	Preoperative	2.15 ± 0.06	2.14 ± 0.04	1.062	0.291
	1 day	2.51 ± 0.10^a^	2.53 ± 0.03^a^	−1.546	0.125
Cobb (°)	Preoperative	16.16 ± 1.72	16.25 ± 1.05	−0.355	0.750
	1 day	7.25 ± 0.52^a^	7.21 ± 0.47^a^	0.434	0.665

Compared with preoperatively.

a *P* < 0.05: The continuous value was given as the mean and the standard deviation.

### 3.5 Comparison of the postoperative secondary indicators

While the manual PVP group demonstrated shorter operative times compared to the robotic-assisted group, the latter showed significantly reduced cement leakage rates (*P* < 0.05). No statistically significant differences were observed between groups regarding cement volume administered, total hospitalization costs, intraoperative blood loss, or postoperative hospital stay duration (*P* > 0.05), see Table 5.

**Table 5:** Secondary indicators after surgery.

Items	Machine group (*n* = 43)	Manual group (*n* = 68)	X^2^/t	*P*
Operative time (min)	48.57 ± 8.27	41.04 ± 9.48	4.029	**0**.**045**
Bone cement (mL)	4.94 ± 0.94	5.28 ± 1.36	−7.338	0.154
Expenses (¥)	10,963.76 ± 2,613.56	12,332.57 ± 5,244.78	−3.431	0.071
Cement leakage (*n*)	5	21	16.378	**<0**.**001**
Bleeding (mL)	3.04 ± 1.44	3.17 ± 1.38	−2.246	0.640
Postoperative hospital stay (d)	4.06 ± 2.13	4.60 ± 1.58	3.856	0.162

Note: Data in bold indicates statistical difference.

### 3.6 Typical cases

#### 3.6.1 Manual group

**Figure 3:**
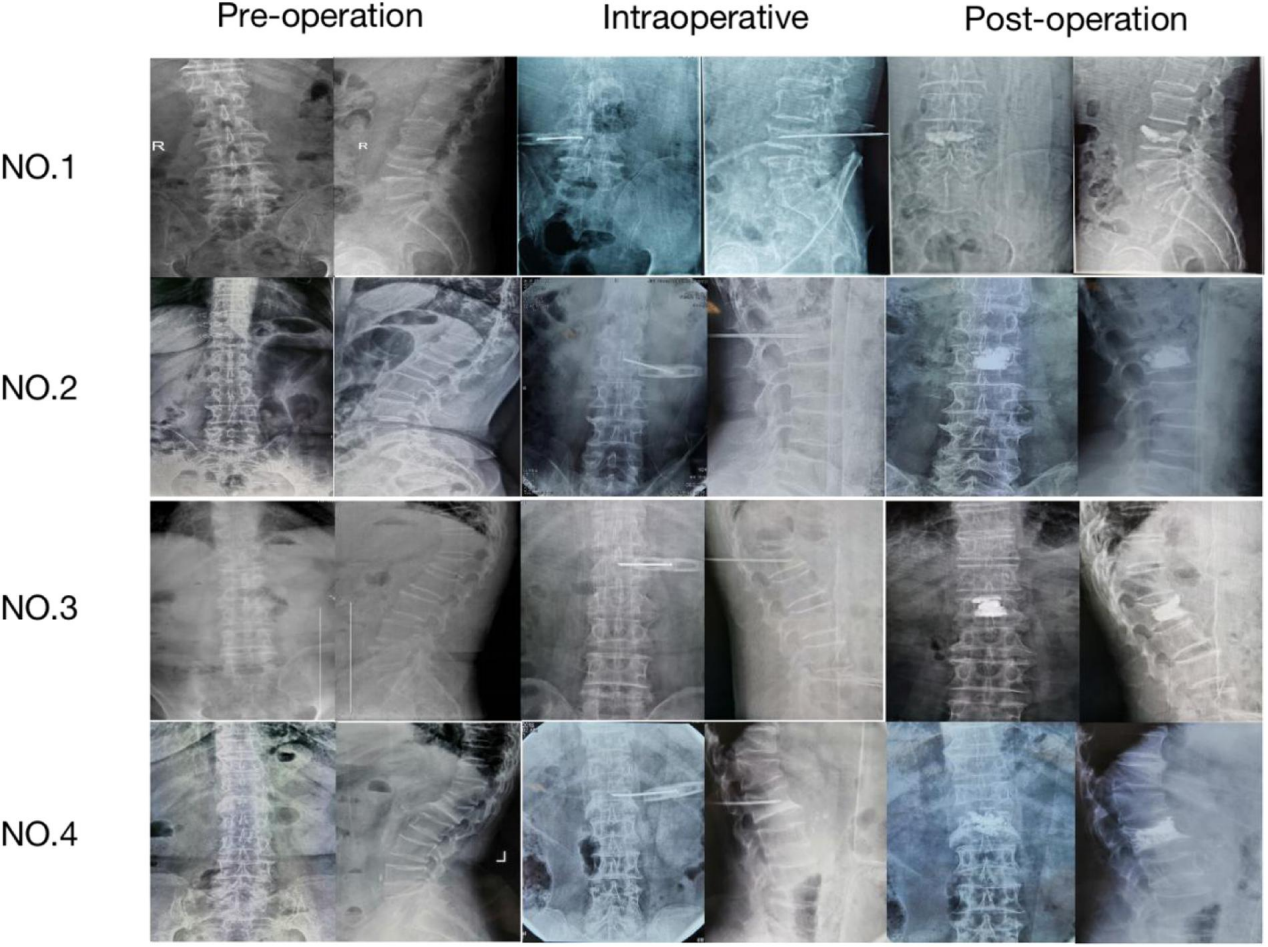
x-ray film of thoracolumbar spine in artificial group (preoperative, intraoperative and postoperative).

NO. 1: male, 70 years old, Chief complaint: Low back pain and activity limitation for 2 months, diagnose: L_4_ compression fracture; NO. 2: female, 64 years old, Chief complaint: low back pain and activity limitation after tumble for 1 week, diagnose: L_2_ compression fracture: NO. 3: male, 83 years old, Chief complaint: low back pain and activity limitation for 10 days, diagnose: L_1_ compression fracture: NO. 4: female, 75 years old, Chief complaint: low back pian and activity limitation for 1 week, diagnose: L_1_ compression fracture (see Figure 3 and Tables 6,7).

**Table 6:** Vertebral body recovery height.

Case	Preoperative vertebral height measurement(cm)			Vertebral height measurement one day after the operation(cm)		
	The height of the anterior edge of the vertebral body	Height of the middle part of the vertebral body	The height of the posterior margin of the vertebral body	The height of the anterior edge of the vertebral body	Height of the middle part of the vertebral body	The height of the posterior margin of the vertebral body
NO. 1	1.41	1.35	2.53	2.52	2.20	2.54
NO. 2	1.77	1.51	2.31	2.12	2.24	2.49
NO. 3	1.64	1.66	2.57	2.03	1.98	2.65
NO. 4	1.71	1.78	2.48	2.40	1.95	2.51
NO. 5	1.52	2.02	2.40	1.84	2.13	2.56
NO. 6	1.95	2.10	2.23	2.16	2.27	2.66
NO. 7	1.44	1.37	2.01	1.98	1.90	2.12
NO. 8	2.01	1.95	2.18	2.19	2.21	2.68

**Table 7:** Spinal Cobb angle.

Case	Preoperative Cobb angle(°)	Cobb angle one day after the operation(°)
NO. 1	16.2	6.9
NO. 2	18.7	8.5
NO. 3	17.5	7.4
NO. 4	17.1	7.2
NO. 5	17.6	7.5
NO. 6	14.4	6.2
NO. 7	18.5	7.7
NO. 8	15.8	6.7

#### 3.6.2 Machine group

**Figure 4:**
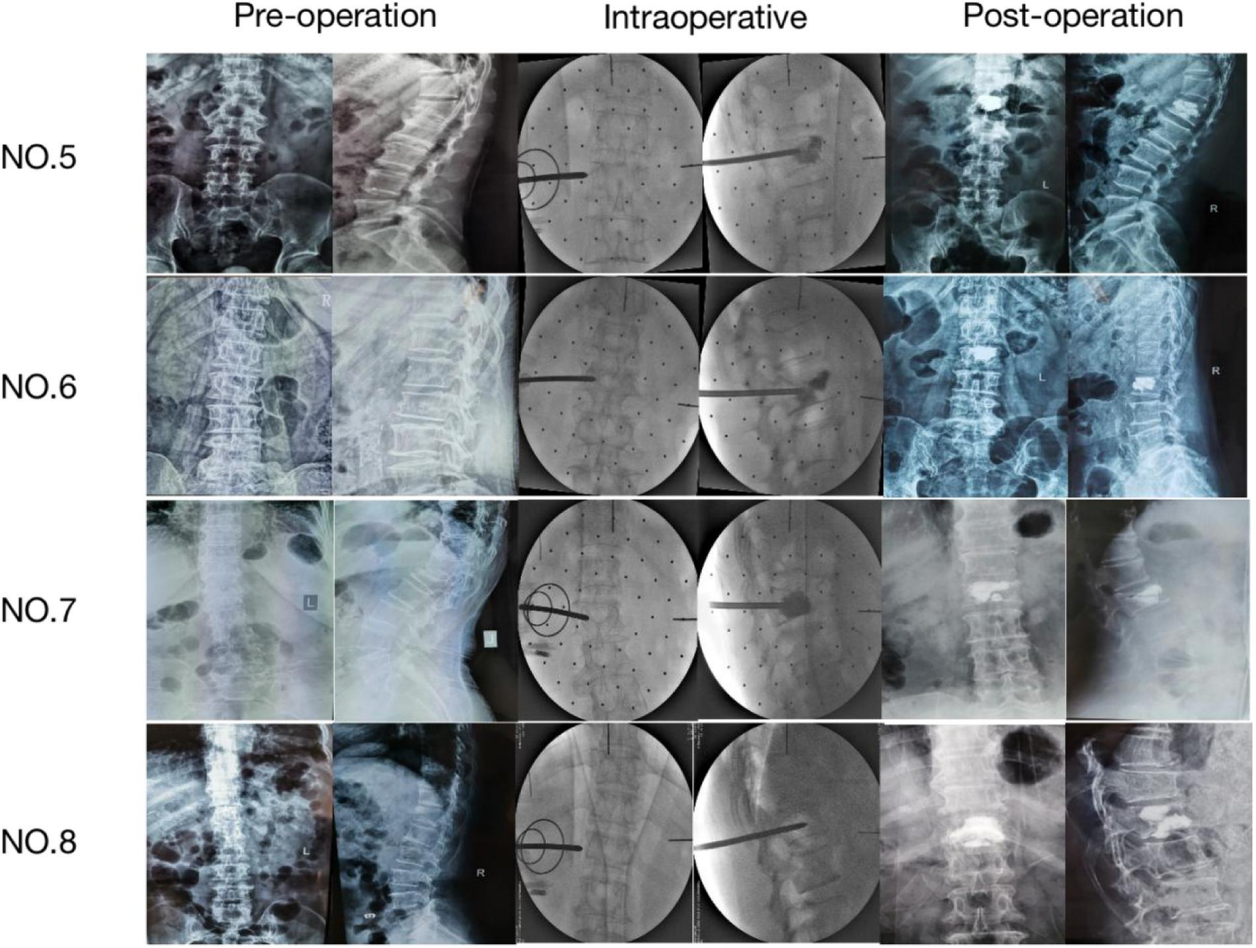
Thoracolumbar spine x-ray film of the machine group (preoperative, intraoperative and postoperative).

NO. 5: male, 57 years old, Chief complaint: Fell down cause low back pain for 3 days, diagnose: L_1_ compression fracture: NO. 6: female, 78 years old, Chief complaint: low back pain and activity limitation for 3 days, diagnose: L_2_ compression fracture: NO. 7: female, 74 years old, Chief complaint: low back pian and activity limitation after tumble for 1 day, diagnose: L_1_ compression fracture; NO. 8: female, 66 years old, Chief complaint: Low back pain and activity limitation for 2 weeks, diagnose: C_12_ compression fracture (see Figure 4 and Tables 6,7).

## 4 Discussion

### 4.1 Summary of the evidence

With increasing population longevity, osteoporosis has emerged as a significant global health concern impacting millions annually (5). The management of osteoporotic spinal fractures, especially vertebral compression fractures, has undergone substantial transformation in the past thirty years, with notable advancements in treatment indications, methodologies, and surgical techniques (6). Surgical intervention for thoracolumbar compression fractures focuses on achieving bony union and anatomical restoration, potentially employing anterior, posterior, or combined approaches that may incorporate decompression, bone grafting, or internal fixation as required (7). Initially developed for hemangioma management, percutaneous vertebroplasty has demonstrated substantial and durable analgesic effects for osteoporotic spinal fractures by restoring mechanical stability (8). This minimally invasive approach has proven particularly effective in providing rapid pain relief from pathological vertebral fractures while preventing additional vertebral collapse and neural compression (9–14). While conventional manual percutaneous vertebroplasty remains the current clinical standard for osteoporotic thoracolumbar compression fractures, this study aims to evaluate robot-assisted vs. manual vertebroplasty, assessing the feasibility of robotic assistance and offering spine surgeons an additional treatment option for this condition.

Robotic assistance effectively reduces operative duration while enhancing procedural safety. Liu et al. (15) demonstrated that computer navigation systems significantly decrease intraoperative C-arm fluoroscopy time, substantially improving radiation safety for both surgical staff and patients. Li et al. (16) found robotic-assisted techniques superior to conventional manual methods in terms of operative time. Peng (17) pointed out that in the early stages, the operative time for robot-assisted surgery was roughly the same as that for traditional manual surgery. This was primarily due to the time required for spinal scanning and the design of the puncture plan before robot navigation. However, as the number of surgical cases increased, the operative time in the robot-assisted group became significantly shorter than that in the traditional group. There are relevant reports (18) indicating that no course can ensure a surgeon's competence in robotics or any related procedures; however, similar to general residency training, it can increase the likelihood of the user becoming proficient with the device. And Chen et al. (19) proposed that the primary reason for the reduction in robot-assisted surgery time compared to traditional manual surgery is the decrease in overall robot usage time. Therefore, as proficiency increases, the robot-assisted surgery time can be shortened. However, conflicting evidence suggests navigation-assisted procedures may prolong surgery, with Lin et al. (20) reporting longer operative times in minimally invasive groups compared to open approaches. Lin et al. (21) noted that many orthopedic surgical robots require extensive preoperative data processing and positioning, complicating the surgical process. Mohamed (22) attributed prolonged operative times to additional requirements for image acquisition, screw trajectory planning, and robotic system positioning. This study's analysis revealed longer operative times for robot-assisted PVP compared to manual PVP, with the authors suggesting this discrepancy with previous findings may stem from surgeons’ initial unfamiliarity with the robotic system (requiring navigation setup time and demonstrating a learning curve) or variability in operators among included cases.

Bone cement leakage represents a crucial determinant of PVP surgical outcomes and constitutes a frequent yet severe complication. This phenomenon can result in cement extravasation into the spinal canal, potentially causing thermal injury to neural structures (23), or embolic events affecting pulmonary and other vital organs. Sun et al. (24) proposed that endplate leakage into intervertebral discs may elevate adjacent vertebral pressure, predisposing to subsequent fractures. Lu et al. (25) further demonstrated that cement-induced disc pressure alterations could deform adjacent vertebral endplates, increasing fracture risk. Research indicates (26) vertebral pressure may increase sixfold with 7 mL cement injection, directly contributing to leakage. The author suggests that variable compression severity in osteoporotic fractures produces diverse fracture patterns with corresponding differences in spinal stability, all significantly influencing leakage likelihood. Posterior leakage due to technical errors may exacerbate pain and carry paralysis risks. This study observed 5 leakage cases in the robotic group vs. 21 in the manual group, demonstrating significantly reduced incidence with robotic assistance. These findings suggest robot-assisted PVP substantially decreases cement leakage risk while maintaining therapeutic efficacy, indicating considerable clinical potential.

### 4.2 Limitations of the article

(1) As this is a single-center study with all data derived from a single medical institution, the demographic characteristics of the study population, clinical protocols, and regional cultural background may be relatively homogeneous. Future research should involve multi-center collaboration to include more diverse and representative samples to validate the conclusions of this study. (2) The follow-up period was relatively short. With a follow-up duration of six months in this study, it may be insufficient to observe the long-term effects of the intervention, late complications, or sustained changes in outcome measures. Future studies should aim to implement longer follow-up periods to assess the long-term robustness of the results. (3) Some outcome measures in this study relied on the subjective judgment of the researchers or patients. Although standardized assessment procedures were adopted and assessors were trained, it remains difficult to completely avoid the influence of measurement biases (such as expectation bias or recall bias). In subsequent studies, employing more objective measurement tools and double-blind assessment methods will help reduce such biases. (4) This trial is a newly initiated clinical experiment with a relatively limited sample size and some imbalance between groups. Although the basic statistical requirements were met, the small sample size may lead to insufficient statistical power, potentially failing to detect clinically significant differences and increasing the risk of false-negative results. Expanding the sample size is an important direction for future research. (5) The indicators included in this study were limited. Subsequent experimental research will continue to refine relevant indicators (such as fluoroscopy frequency, radiation dose, etc.).

### 4.3 Conclusion

Manual percutaneous vertebroplasty demonstrates superior operative time compared to robot-assisted percutaneous vertebroplasty. However, the robotic approach offers the advantage of reduced cement leakage, which enhances procedural safety and decreases postoperative complications to some degree. Furthermore, as surgeons gain proficiency with the robotic system, operative time can be further reduced. This technology warrants continued refinement and represents a promising direction for future development.

## Data Availability

The raw data supporting the conclusions of this article will be made available by the authors, without undue reservation.
