# Correction: A comparison of short-term outcomes between robot-assisted percutaneous vertebroplasty and manual percutaneous vertebroplasty in the treatment of osteoporotic thoracolumbar vertebral compression fractures

**DOI:** 10.3389/fsurg.2025.1724871

**Published:** 2025-10-28

**Authors:** Hang Lin, Kun Li, Zhibin Zhang, Abuduwupuer Haibier, Tuerhongjiang Abudurexiti

**Affiliations:** 1 Spinal Surgery, Sixth Afliated Hospital of Xinjiang Medical University, Urumqi, Xinjiang Uygur, China; 2 Xinjiang Medical University, Urumqi, Xinjiang Uygur, China

**Keywords:** robot, robot-assisted navigation, percutaneous vertebroplasty, osteoporotic thoracolumbar vertebral compression fracture, cement leakage

Authors **Hang Lin, Kun Li, Zhibin Zhang**, and **Abuduwupuer Haibier** were erroneously omitted as equal contributing authors.

The original version of this article has been updated.

